# Impact of initial lip competence on the outcome of class II functional appliances therapy

**DOI:** 10.1007/s00784-024-05511-x

**Published:** 2024-01-30

**Authors:** Janine Sambale, Anahita Jablonski-Momeni, Heike Maria Korbmacher-Steiner

**Affiliations:** Department of Orthodontics, Clinic of Dentistry, Philipps-University Marburg, Georg-Voigt-Str. 3, 35039 Marburg, Germany

**Keywords:** Orofacial dysfunction, Lip incompetence, Lip competence, Class II division 1, Functional jaw orthopedics, Functional appliance

## Abstract

**Objectives:**

The aim of this prospective clinical study was to evaluate the impact of initial lip position on class II functional appliance therapy.

**Materials and methods:**

In total, 34 class II division 1 patients (23 females, 19 males; mean age 12.4 ± 0.9 years) that met the inclusion criteria (> ½ class II molar relationship, overjet > 6 mm, ANB > 4°, neutral or horizontal growth pattern, cervical vertebral maturation stage (CVMS) II – III, mean wear-time > 10 h/day) were consecutively divided into two groups (lip incompetence (LI); lip competence (LC)). All patients were treated with the Sander bite jumping appliance (BJA). Wear time was microelectronically measured. Lateral cephalograms were taken at the beginning (T0) and after 1 year of treatment (T1). An untreated class II group served as a control (CG). Inter-group comparisons were determined with Mann–Whitney *U* tests for independent samples.

**Results:**

Significant skeletal treatment effects were found in both treated groups when compared to the CG with significantly more pronounced mandibular skeletal effects in the LI than in the LC group (mandibular base length *p* < 0.001, composite mandibular base length *p* < 0.001, condylar head growth *p* = 0.002, co-pg *p* < 0.00, go-pg *p* = 0.003, reduction of the ANB angle *p* = 0.009, and Wits appraisal *p* < 0.001).

**Conclusion:**

The more pronounced mandibular effects in the LI group were composed of the functional orthopedic effect plus harmonization of the lip competence.

**Clinical relevance:**

Functional harmonization of lip incompetence with BJA enhances mandibular growth stimulation. Lip incompetence seems to impede mandibular growth and its harmonization seems to be a preventive approach.

## Introduction 

In Europe, the prevalence of class II anomalies varies between 19.3 and 30% [1]. The etiology is often a multifactorial, partly polygenetic process [2]. Nevertheless, the hereditary predisposition can be modified by epigenetic factors such as dysfunctions and muscle imbalance. Functional jaw orthopedics (FJO) are proved to be very effective in low-angle cases and neutral growth pattern in harmonizing the sagittal discrepancy by skeletal and dental effects [3–5]. However, treatment timing plays an important role. The most effective timing for FJO of class II anomaly is shown to be during or slightly after the onset of the puberal growth spurt II [6, 7]. The orthopedic advancement of a retrognathic mandible with FJO was also confirmed by other studies [5, 7–10] and the achieved mandibular position remained stable in the long term [4, 11–13].

In the case of severe class II anomaly, a two-stage treatment with functional pre-treatment is advisable as an increased overjet with inadequate mouth closure is associated with a higher risk of a more severe incisor trauma [14–16]. Moreover, according to the functional matrix theory, muscle imbalance could lead to growth restriction [17]. Ignoring persistent orofacial dysfunctions often results in relapse [17–20]. The main function of the lips, and therefore lip competence, is controlled by the orbicularis oris muscle, which is often weak in class II division 1 anomalies, while at the same time a hyperactivity of the mentalis muscle persists [21].

A prospective clinical trial with classification of the patient population based on initial lip competence and an investigation due to possible dentoskeletal differences under class II treatment with FJO has not yet been conducted so far. Thus, the aim of the present study was to investigate the impact of the epigenetic factor “lip incompetence” on the outcome of functional appliance therapy. The question was raised whether patients with initial lip incompetence show different reactions regarding orthopedic and dental effects under functional appliance treatment than patients with lip competence, when similar initial conditions in terms of skeletal class II morphology, growth pattern, treatment timing, and compliance exist.

## Subjects and methods

### Study population

**Fig. 1:**
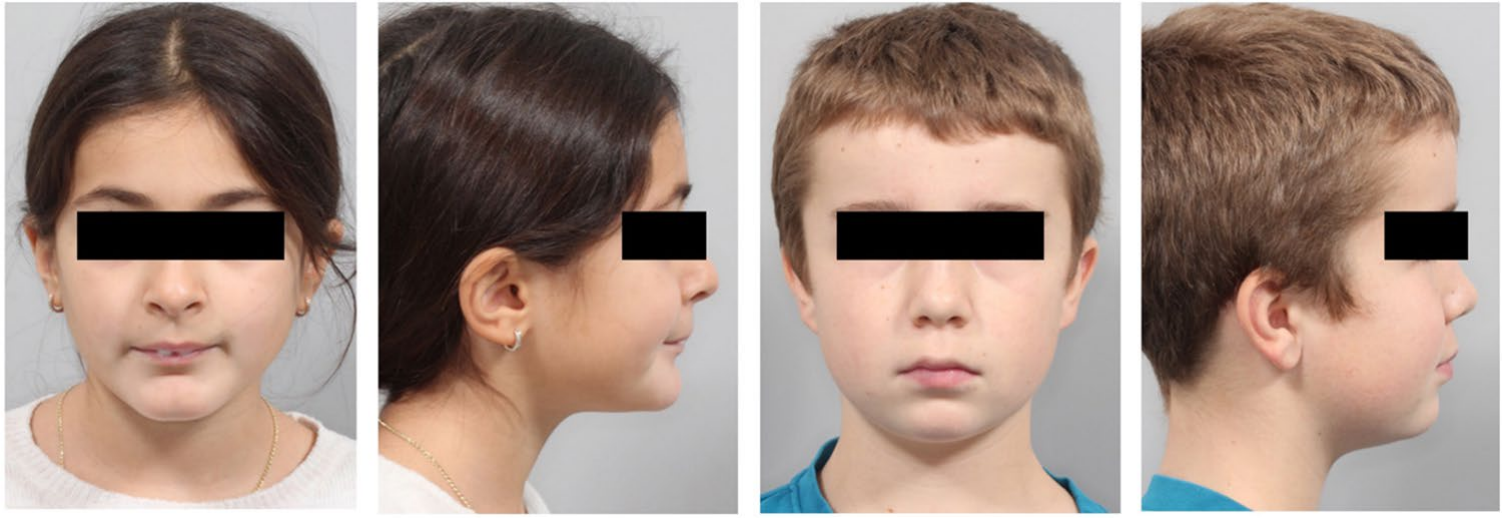
Representative patient examples for patients with initial lip incompetence (LI): **a** enface and **b** profile and with initial lip competence (LC) **c** enface and **d** profile

Ethical approval was obtained from the Ethics Committee of Philipps-University Marburg (reference no. 145/19) and the study was registered in the German Clinical Trials Register (DRKS00021090, date of registry: 12 March 2020). A sample size calculation was performed (MedCalc Software, version 22.009, Ostend, Belgium) based on preliminary (unpublished) data. A number of 9 patients was calculated for each treatment group (Power 0.95, *α* = 0.05), assuming a mean difference of 1.5 and a standard deviation of 0.8 in each group (Wits). A drop-out number of 20% was added in each group. In total, 11 patients should be included in each group. After that class II division 1 patients were recruited and divided into two equal-sized groups based on initial lip competence. In total 42 patients were recruited at the Department of Orthodontics, Institute of Dentistry, University of Marburg, Germany, between 2020 and 2022. The inclusion criteria were as follows: more than a half premolars width class II molar relationship (i.e., > 3.5 mm), overjet > 6 mm, late mixed dentition, ANB > 4°, sum of Björk polygon angles with neutral (396 ± 5°) or horizontal (< 391°) growth pattern, and cervical vertebral maturation stage (CVMS) II – III [22]. The exclusion criteria were lack of patient’s willingness to sign an informed consent form, craniofacial anomalies, vertical growth pattern (sum of Björk polygon angles: > 401°), tooth extraction, previous or additional orthodontic therapy, rheumatic disorders, and bone metabolism-altering medications. The patients did not statistically differ regarding the chronological (mean 12.4 ± 0.9 years) and skeletal age (CVMS) as well as the severity of skeletal class II morphology (ANB, Wits, growth pattern). Two orthodontics (JS and HKS) involved in the recruitment process and in the treatment were calibrated regarding the methodological and clinical procedures prior to the start of the study. The assessment of the clinical initial lip competence was performed independently by each examiner at the dental chair and subsequently checked for agreement. The interrater reliability was evaluated using *κ*-statistics and revealed perfect agreement (*κ* = 0.97) [23]. The patients were recruited in the order of their initial assessments and divided into two equal-sized groups based on the initial lip competence. They were allocated to either a lip incompetence (LI; *n* = 21) or lip competence group (LC; *n* = 21) (see Fig. 1a–d). The treated groups were compared with an untreated control group (CG) published by Baccetti et al. [7]. The CG consisted of 14 subjects (seven females and seven males) with a skeletal age of CVMS analysis II–III and a mean observation period of 1.3 ± 0.5 years.

### Appliance and treatment protocol

**Fig. 2:**
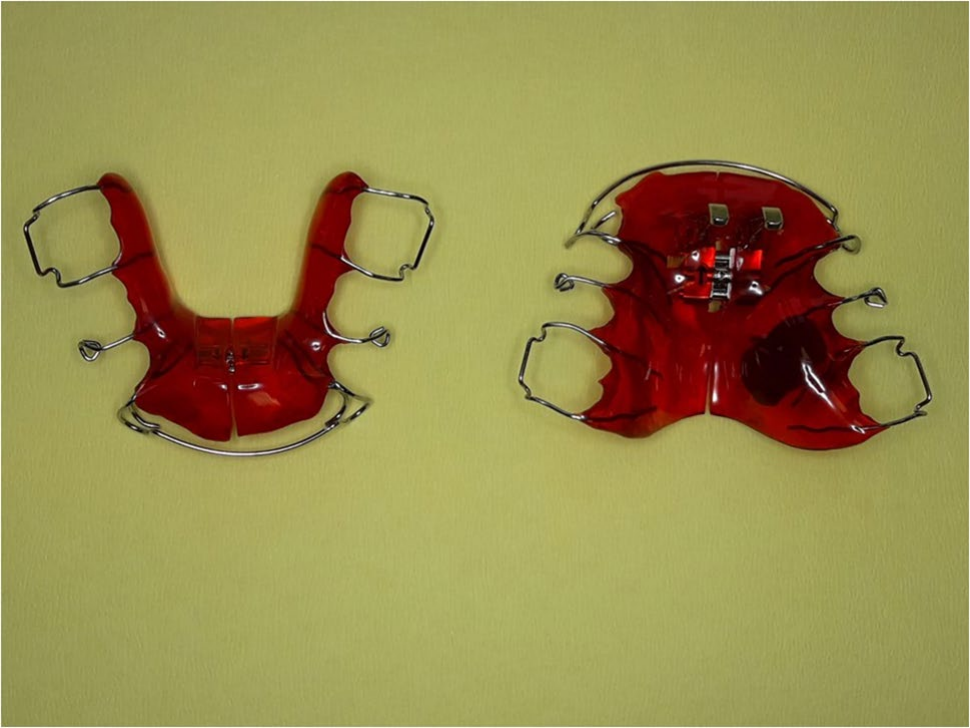
Example of the construction of the Sander bite jumping appliance (BJA) with incorporated temperature-sensitive microsensor (TheraMon®, MC Technology GmbH, Austria) in the upper plate

**Fig. 3:**
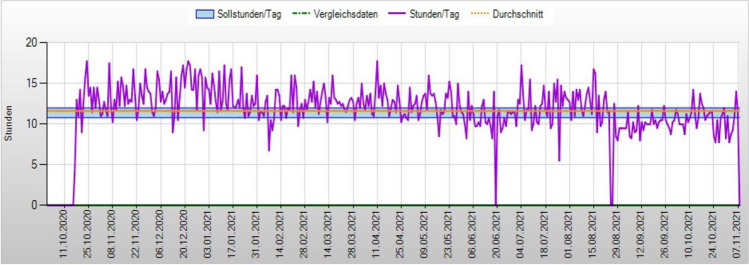
Example of a patient’s 1-year wear-time documentation between T0 (2020) and T1 (2021). The blue area represents the target wear time. The orange horizontal line shows the mean wear time of the appliance (11.35 h/day), while the violet line shows the exact daily wear time

The patients were treated using the Sander bite jumping appliance (BJA). The appliance was constructed as described by Gazzani et al. [5]. The expansion screw in the upper jaw was activated (one turn = 0.25 mm per week) in cases of initial transversal discrepancy. The expansion screw in the lower jaw was activated (one turn = 0.25 mm per month) in cases of initial lingual tipping of the lower molars. Activating the lower expansion screw led to leveling the curve of Wilson. The therapeutically desired jaw relation was three-dimensionally registered with a wax construction bite with one-step mandibular advancement. In the sagittal plane, the mandible was positioned in super-class I molar relationship. In the transversal plane, a gnathic midline shift was corrected and in the vertical plane, the mandible was positioned with a 2-mm frontal vertical opening. Activation of the transversal screws affected only incisor position if it was desired, otherwise the labial bow was deactivated during the expansion period. The bite registration, establishing a super class I molar relationship, determined whether the upper labial bow needed activation or deactivation and the extent to which upper incisors had to be reclined to achieve a physiological overjet of 2 mm. In every patient, lingual reduction of the lower plate was performed, and the labial bow was activated to prevent significant dental side effects, such as proclination of the lower incisors. The patients were motivated to wear the appliance more than 12 h/24 h. To measure the wear time objectively, a temperature-sensitive microsensor (TheraMon®, MC Technology GmbH, Austria) was polymerized into the upper plate (Fig. 2) [24]. The patients were seen every 6 weeks and the objective wear time was registered with the TheraMon® pen. The documentation of wear time was presented to the patients, and they were motivated to maintain the affordable compliance (Fig. 3). An overall mean wear time of less than 10 h/24 h led to an exclusion of the study. After the end of the treatment (T1), a class I molar relationship or super class I molar relationship was achieved and the mandible could no longer be pulled backwards. The mean treatment duration was 1.1 ± 0.1 years.

### Cephalometric analysis

Exposure values for the lateral cephalograms (PLANMECA, ProMax) were set to 66–68 kV and 5 mA depending on the patient’s size. Patients were secured with a nasal rest to Nasion (N) and head inclination was adjusted according to the Frankfurt horizontal plane. Lateral cephalograms were taken in centric occlusion with lips in the resting position at T0 and T1 and were standardized using a magnification factor. Both lateral cephalograms were conducted as part of the routine treatment of the functional orthopedic treatment. The cephalograms were blinded to the patients’ name and allocation, and were analyzed with a digitizing software (Ivoris analyze version 8.2.62.130) by the author (JS). To ensure quality of data assessment, intra- and interexaminer agreements (*κ*-values) were evaluated. Two orthodontists had been extensively trained in cephalometric analysis by one author (JS). Two randomized cephalograms (T0 and T1) were examined together, while 20 (T0 and T1) were independently analyzed by the two orthodontists. According to the intraexaminer consistency and reproducibility, one author (JS) reanalyzed the cephalograms 30 days later. Method errors varied from 0.17 to 0.76 mm. Intra- and interexaminer reliability values ranged *κ* = 0.92–0.96, indicating perfect agreement [23].

The modified Pancherz analysis [25] by Baccetti et al. [7] was applied using the following variables with additional measurements represented in Table 1.

**Table 1:** Definition of the skeletal and dental variables

A/OLp	Sagittal position of the maxillary base
pg/OLp	Sagittal position of the mandibular base
co/OLp	Sagittal position of the condylar head
pg/OLp + co/OLp	Composite mandibular length
is/OLp—ii/OLp	Overjet
ms/OLp—mi/OLp	Molar relation (a positive value indicates a distal, a negative value indicates a mesial molar relationship)
is/OLp—A/OLp	Sagittal position of the maxillary central incisor within the maxilla
ii/OLp—pg/OLp	Sagittal position of the mandibular central incisor within the mandible
ms/OLp—A/OLp	Sagittal position of the maxillary permanent first molar within the maxilla
mi/OLp—pg/OLp	Sagittal position of the mandibular permanent fist molar within the mandible
co-pg	Total mandibular length
co-go	Mandibular ramus height
go-pg	Mandibular body length
nl/T-FMN line	Maxillary plane angle
ml/T-FMN line	Mandibular plane angle
nl-ml	Interbase relation
Additional measurements	
ANB Wits appraisal Sum of Björk polygon angles (Sum of saddle, articular, and gonial angles)	

*OLp*, occlusal line perpendicular

### Statistical analysis

Arithmetic mean (M) and standard deviation (SD) were evaluated for all variables. The Shapiro–Wilk test was performed on all variables to test for normality of distribution. To determine intergroup differences concerning dental and skeletal age such as severity of class II at T0, the Mann–Whitney *U* test was used. The changes between T1 and T0 of all measurements were calculated and inter-group comparisons (LI/control, LC/control, LI/LC) were determined with Mann–Whitney *U* tests for independent samples. A *p* value of ≤ 0.05 was considered statistically significant. To assess the inter- and intrarater reliability, Kappa statistics were determined.

## Results

### Comparison of starting forms

From the 42 patients, six patients were excluded from the study due to non-adherence (mean wearing time < 10 h/24 h), while two patients canceled the entire orthodontic treatment themselves. In total, data of 34 patients could be statistically analyzed. Both groups included 17 patients with 10 females and seven males in the LI group and 12 females and five males in the LC group. At T0, there were no significant differences in terms of gender distribution (*p* = 0.29), skeletal (*p* = 0.42), and chronological mean age (*p* = 0.12) between LI and LC groups. The mean wearing time was 10.74 ± 0.73 h/24 h in the LI group and 11.01 ± 0.82 h/24 h in the LC group and was not statistically significant (*p* = 0.42). Significant differences at T0 were only noted for the dentoalveolar variables overjet (*p* < 0.001) and is/OLp-A/OLp (*p* = 0.03) which showed significantly higher values in the LI group.

### Treatment effects in the LC group (Table 2, Fig. 4)

Treatment with the BJA led to an overjet correction of 4.8 mm and a molar relation correction of 3.9 mm. The skeletal contribution to overjet correction was 53%. Skeletal mandibular changes (+ 1.8 mm) were higher than maxillary changes (–0.7 mm) showing a significantly greater mandibular base measurement (*p* = 0.037) than the control group. Functional jaw orthopedics induced a significant backward displacement of the condylar head (co/OLp, *p* < 0.001), significant increases in total mandibular length (co-pg, *p* = 0.005), and ramus height (co-go, *p* = 0.002) when compared to the CG. No significant differences were found for the composite mandibular base length (pg/OLp + co/OLp) and body length (go-pg). Maxillary growth restriction was also significantly different (*p* = 0.03) compared to the CG. No significant differences were found regarding vertical skeletal relationships. The dentoalveolar component of overjet correction was only due to significantly mandibular incisor proclination (*p* < 0.001). The minor component of upper incisor retrusion (– 0.24 mm) was covered by sagittal alveolar remodeling of the maxilla (– 0.45 mm) during growth. Skeletal (54%) and dental (46%) components contributing to molar relation correction were almost similar to overjet correction. The dentoalveolar components were in equal parts composed by upper molar distalization (– 1 mm) and lower molar mesialization (+ 0.8 mm) without significant differences when compared with the control subjects.

### Treatment effects in the LI group (Table 2, Fig. 4)

The BJA treatment of patients with lip incompetence produced an overjet and molar relation correction of 7.1 mm. The skeletal contribution to overjet correction was dominant (73%) and mainly resulted from high skeletal mandibular base length changes (+ 4.4 mm) with statistically significant differences compared to the CG (*p* < 0.001). Compared to the CG, all other skeletal mandibular measurements showed statistically significant increases: composite mandibular length (pg/OLp + co/OLp, *p* < 0.001), total mandibular length (co-pg, *p* < 0.001), body length (go-pg, *p* = 0.001), and ramus height (co-go, *p* < 0.001). Functional jaw orthopedics induced a significant backward displacement of the condylar head (co/OLp, *p* < 0.001). In contrast to the high increase of mandibular length changes, maxillary restriction was minor (– 0.8 mm), but significantly different when compared to the control (*p* = 0.006). No significant differences were found for vertical skeletal relationships. The dentoalveolar component of overjet correction was driven by significant maxillary incisor retrusion (*p* < 0.05) and mandibular incisor protrusion (*p* < 0.001). Skeletal (71%) and dental (29%) components contributing to molar relation correction were similar to overjet correction. Upper molar distal movement (+ 1.4 mm) was more than twice as large as lower molar mesial movement (+ 0.6 mm). Upper and lower molar movements were found to be not statistically significant.

### Inter-group comparison LI/LC group (Table 2, Fig. 4)

FJO of patients with initial lip incompetence compared to patients with initial lip competence led to significant higher skeletal changes (LI 73%; LC 54%). The LI group showed a three-times higher skeletal mandibular contribution (+ 4.4 mm; + 4.3 mm) due to overjet and molar relation correction than the LC group (+ 1.8 mm versus + 1.4 mm). Statistically significant greater values were found regarding mandibular base length (pg/OLp, *p* < 0.001), composite mandibular base length (pg/OLp + co/OLp, *p* < 0.001), total mandibular length (co-pg, *p* < 0.001), and mandibular body length (go-pg, *p* = 0.003) between both treated groups. There was also a significant greater backward displacement of the condylar head (co/OLp) in the LI group compared to the LC group (*p* = 0.002). The reductions of ANB angle (*p* = 0.009) and Wits appraisal (*p* < 0.001) were also significantly greater. Mandibular ramus height (co-go), maxillary base (A/OLp), and vertical skeletal measurements (nl/FMN-T, ml/FMN-T, nl-ml) did not significantly differ between the two treated groups. For the dentoalveolar cephalometric aspects, the maxillary incisors of the LI group showed a significantly higher retrusion (*p* < 0.05) while the mandibular incisors of the LC group showed a significantly higher protrusion (*p* < 0.001). Regarding the position of the maxillary and mandibular molars, there was no statistically significant difference between both treated groups.

**Table 2:** Cephalometric changes between pre- and post-treatment (T1-T0), multiple comparison, and “treatment effect” between the three groups

Variable	Lip incompetence (LI)		Lip competence (LC)		Control		Multiple comparison (*p* value)			Group difference (“treatment effect”)	
	Mean	SD	Mean	SD	Mean	SD	LI/ Control	LC/ Control	LI/ LC	LI/ Control	LC/ Control
Overjet (is/OLp minus ii/OLp)	− 7.20	0.96	− 4.93	1.27	− 0.12	1.39	< 0.001	< 0.001	< 0.001	− 7.08	− 4.81
Molar relation (ms/OLp minus mi/OLp)	− 7.35	0.69	− 4.03	1.34	− 0.13	0.56	< 0.001	< 0.001	< 0.001	− 7.22	− 3.90
Maxillary base (A point/OLp)	− 0.21	0.56	− 0.05	0.67	+ 0.56	0.86	0.006	0.03	NS	− 0.77	− 0.61
Mandibular base (pg/OLp)	+ 5.25	1.93	+ 2.72	1.02	+ 0.90	2.14	< 0.001	0.037	< 0.001	+ 4.35	+ 1.82
Condylar head (co/OLp)	− 1.62	1.16	− 0.49	0.68	− 0.20	1.30	< 0.001	< 0.001	0.002	− 1.42	− 0.29
Composite mandibular length (pg/OLp + co/OLp)	+ 6.46	1.73	+ 2.17	1.34	+ 1.11	2.25	< 0.001	NS	< 0.001	+ 5.35	+ 1.06
Maxillary incisor (is/OLp minus A point/OLp)	− 0.80	0.76	− 0.24	0.89	− 0.45	1.73	< 0.05	NS	< 0.05	− 0.35	+ 0.21
Mandibular incisor (ii/OLp minus pg/OLp)	+ 0.90	0.69	+ 1.84	0.55	− 0.68	1.41	< 0.001	< 0.001	< 0.001	+ 1.58	+ 2.52
Maxillary molar (ms/OLp minus A point/OLp)	− 1.36	2.52	− 0.96	1.53	+ 0.03	1.38	NS	NS	NS	− 1.39	− 0.99
Mandibular molar (mi/OLp minus pg/OLp)	+ 0.44	0.43	+ 0.64	0.37	− 0.18	1.51	NS	NS	NS	+ 0.62	+ 0.82
co-pg (mm)	+ 6.68	3.25	+ 3.78	1.18	+ 2.54	1.01	< 0.001	0.005	< 0.001	+ 4.14	+ 1.24
co-go (mm)	+ 5.05	3.44	+ 3.25	1.74	+ 1.25	1.45	< 0.001	0.002	NS	+ 3.80	+ 2.0
go-pg (mm)	+ 4.85	3.14	+ 2.11	1.54	+ 1.57	1.14	0.001	NS	0.003	+ 3.28	+ 0.54
nl/FMN-T line (°)	+ 0.57	0.67	+ 0.24	1.00	+ 0.52	1.38	NS	NS	NS	+ 0.05	− 0.28
ml/FMN-T line (°)	+ 0.71	1.32	+ 0.85	1.34	− 0.30	1.59	NS	NS	NS	+ 1.01	+ 1.15
nl-ml (°)	+ 0.16	2.10	+ 0.83	2.27	− 0.82	0.89	NS	NS	NS	+ 0.98	+ 1.65
ANB (°)	− 1.86	0.56	− 1.35	0.51	/	/	/	/	0.009	/	/
Wits (mm)	− 3.39	0.88	− 2.22	0.75	/	/	/	/	< 0.001	/	/
Sum of Björk polygon angles	+ 1.51	0.32	+ 1.49	0.35	/	/	/	/	NS	/	/

*SD* standard deviation, *NS* non-significant

**Fig. 4:**
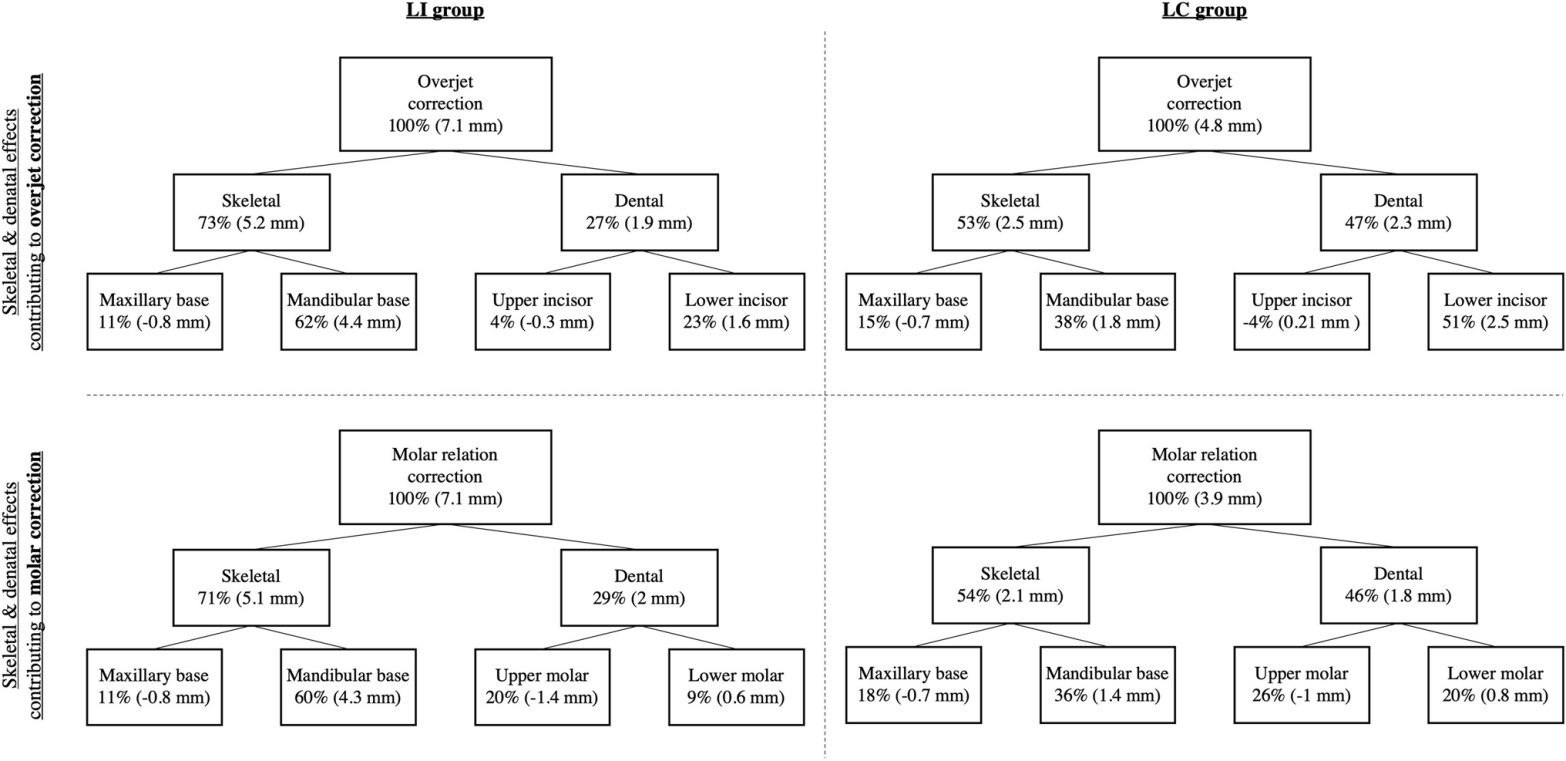
Graphical illustration of maxillary and mandibular skeletal and dental changes (“treatment effect”) contributing to overjet and molar relation correction between the treated groups (left: LI group, right: LC: group)

## Discussion

This investigation was the first prospective clinical trial with differentiation of class II division 1 patients based on initial lip competence or incompetence. Although many studies investigated skeletal and dentoalveolar changes under functional orthopedic treatment, no previous study investigated dentoskeletal changes of patients with class II treatment regarding initial lip incompetence. Because of ethical issues, it was not possible to compare our treated groups with a contemporary untreated class II group for long-term observation. Long-term observation during the pubertal growth spurt suggests that the prepubertal advancement of the mandible, leading to the establishment of a class I molar relationship through functional treatment, is missed. For ethical reasons, a historical control group, which may be considered a limitation [26], was used in the current study. A recent study revealed that trials using historical controls demonstrated smaller treatment effects in comparison to trials using concurrent controls [27]. In simpler terms, it appears that historical controls do not seem to magnify treatment effects in comparison to concurrent controls. Further studies assessing lip competence versus lip incompetence in class II patients continued follow-up observations after functional jaw orthopedics are necessary to evaluate long-term stability of lip competence.

At the beginning of the orthopedic treatment, there were no significant differences in terms of gender distribution, chronological age, skeletal variables, and wear time, but significantly higher values for the dentoalveolar variables such as overjet and upper incisor proclination in the LI group related to abnormal lip posture. Patients with vertical growth pattern were not included because a long-face subject with clockwise rotation of the mandible would have a reduced sagittal advancement of the mandible after treatment compared to low-angle patients or patients with neutral jaw angles [28]. Due to ideal neutral or horizontal growth pattern in our treated groups, bite elevation was not necessary and vertical dimensions remained unchanged.

The treatment outcome of the LC group compared to the untreated control group was not unexpected and showed similar dentoskeletal results as already described in literature [7, 28]. The LC group showed an efficient overjet or molar relation correction of 53% or 54% skeletal effects compared to the untreated control group. The skeletal effects were mainly related to mandibular skeletal effects, but there was also a slightly inhibitory effect on the sagittal growth of the maxilla compared to the untreated group. Maxillary growth restriction of functional jaw orthopedics (FJO) has been reported as a consequence of reciprocal forces with a posterior directed force to the maxilla when the mandible is pushed forward [29]. Several studies have already proven this effect [30–32], while others did not [3, 33, 34]. Furthermore, the LC group showed the typical dentoalveolar side effects evoked by functional class II appliances with significant protrusion of the lower incisors, slight retrusion of the upper incisors, and distalization of the upper and mesialization of the lower molars [8, 9, 11, 13]. Distalization of the upper molars with reciprocal forces regarding mesialization of the lower molars related to the “headgear effect” has been reported in several studies earlier [11, 13].

In contrast, patients with initial lip incompetence showed significant higher skeletal effects than the LC group with 73% or 71% skeletal contribution to overjet or molar relation correction. The occlusal changes were also mainly related to skeletal mandibular effects with an increase of mandibular base length (pg/OLp) in the LI group (4.35 mm) of more than twice than that of the LC group (1.82 mm).

The different treatment outcomes of the two treated groups underline the impact of lip incompetence in the outcome of functional orthopedic treatment. Several authors proved that functional appliance treatment contributes to functional harmonization of an abnormal muscle function such as lip incompetence [35–38]. Yang et al. reported that orofacial myofunctional therapy in patients with lip incompetence could effectively improve lip strength and optimize jaw relationship due to forward movement of the mandible. Functional treatment has been proven to establish a new neuromuscular pattern and to correct abnormal muscle function [37]. In our study sample, both treated groups showed lip competence after functional orthopedic treatment. The greater forward movement of the mandible in the LI group was based not only on significantly greater mandibular base length but also on greater increase in total mandibular length (co-pg), mandibular body length (go-pg), and mandibular ramus height (co-go). The greater additional growth of the mandible was associated with significantly greater changes in the mandibular condyle head (co/OLp). The LI group showed significantly more backward growth modification than the LC group. This growth phenomenon was already described as “posterior mandibular morphogenetic rotation,” which is a biological phenomenon leading to a higher increase in total mandibular length [39]. In both groups, the construction bite was identically taken and pushed the mandible into the desired forward direction, which led to increased bone apposition at the superior posterior side of the condyle and posterior side of the ramus. The bone apposition during functional orthopedic treatment has been reported in experimental animal and clinical studies [40, 41]. The fact that the LC group showed the expected treatment outcome after FJO as already reported in literature, whereas the LI group revealed significantly more pronounced mandibular reactions suggests an additional growth stimulus after growth restriction due to abnormal lip posture. This might be interpreted as a “rebound effect” in the sense of “catching up” earlier missed growth. It has been already reported that abnormal orofacial function in the period of growth and development causes morphological anomalies of the craniofacial complex. The morphogenetic shape and mineralization of the jawbone are also related to epigenetic factors and undergo gradual changes in response to external influences like an abnormal orbicularis oris muscle function. These external effects on dentition and jaws are related to the mode, frequency, and duration of an abnormal muscle function [42]. Forces from the tissues in the passive resting position such an abnormal lower lip posture are more important than forces exerted on the teeth during active functions such as swallowing. Forces that act during function are of short duration, but forces like abnormal lip posture operating more than 4 to 6 h per day can lead to unwanted changes of incisor inclination and growth restriction [43].

In the context of form and functional relations, mouth posture plays a crucial role in both diagnosis and therapy. This concept was recognized by Fränkel [44] who first described a threefold mouth closure consisting of labial, linguopalatal, and velolingual closure. Attaining a balance of forces contributes to undisturbed growth with a significant preventive role in adulthood [45].

Therefore, the two-stage treatment with functional pre-treatment plays a crucial aspect of ensuring oral health of patients and preventing growth restriction. According to a recent editorial by Paglia, diagnosing, monitoring, and intervention should take place at different age stages: during age 0–3 years the focus is recommended to lie on breastfeeding monitoring muscle weakness and promoting physiological growth through dietary and lifestyle choices; during age 4–6 years attention should be given to physiological growth development. Interception and correction of bad habits like abnormal lip posture are essential to avoid growth restriction and to ensure physiological growth during this period [17]. Promoting awareness of early signs of muscle imbalance and lip incompetence plays a key role for undisturbed growth of the craniofacial complex [46].

The results of our study thus underline the importance of the two-stage treatment with functional harmonization of lip incompetence avoiding mandibular growth restriction due to abnormal lip posture. These assumptions should be confirmed on younger patients in early mixed dentition to clarify the impact of lip incompetence as an epigenetic factor in the development of the mandible. Changing lifestyle behaviors in the childhood such as increased screen time behavior followed by sleep deprivation with consequently reduced physical activity and increased prevalence of obesity over the last decades [47, 48] will support bad posture and weak muscle tonicity. A high correlation between weaker body posture, muscle tonus, and orofacial dysfunctions has already been reported [49]. Further prospective clinical trials are necessary to assess the impact of earlier functional harmonization among patients with epigenetic factors such as lip incompetence.

## Conclusion

- Class II division 1 patients with and without initial lip competence benefit both from skeletal class II treatment when compared to untreated class II patients.
- FJO among class II.1 patients with initial lip incompetence results in more pronounced favorable skeletal effects than in patients with initial lip competence.
- The more pronounced skeletal treatment results among the patients with initial lip incompetence suggest that the perioral muscle pressure of the lower lip leads to an unwanted restriction of the lower jaw, which is abandoned by the functional appliance therapy.
- Our results underline the importance of treatment strategies focusing on lip competence.
